# Supplementary material for: “It’s okay because I’m just driving”: an exploration of self-reported mobile phone use among Mexican drivers
Source: PeerJ. 2024 Feb 23;12:e16899. doi: 10.7717/peerj.16899 (PMC10896083; doi:10.7717/peerj.16899)
Supplement: Supplemental Information 1 — The English version of the root questionnaire used to conduct the research. [file peerj-12-16899-s001.pdf]

Appendix 1.
Questionnaire structure (draft - researcher form)

Section I.

1- Can you tell me your city of provenance? ________________________

2- Can you tell me your age?_________

3- Sex:

- ☐ Man
- ☐ Woman
- ☐ Other
- ☐ Prefer not to say

4- Do you have a driver's license? (Filter question)

- ☐ Yes
- ☐ No

5- Do you drive at least once a week? (Filter question)

- ☐ Yes
- ☐ No

Section II.

6- To what degree do you consider the use of cell phones while driving to be risky?, on a scale of 1 to 5 (where 1 is not at all and 5 is high risk)

Not at all 1	2	3	4	5 High risk

7- Could you please indicate how often you use your cell phone while driving in the last 30 days? on a scale of 0 to 4 (where 0 is never and 4 is always I drive)

Never 0	1	2	3	4 Always I drive

Have you used your cell phone in the following traffic situations in the last 30 days?

	Yes	No
8- Stopping at a traffic light		
9- Driving in intermittent traffic		
10- Driving at low speed		
11- Driving faster than 25 miles or 40 kilometers per hour		

12- Why do you use your cell phone while driving in the last 30 days? (you can indicate more than one option)

- ☐ Voice calling
- ☐ Reading texts
- ☐ Composing texts or texting
- ☐ Map browsing
- ☐ Social networking
- ☐ Reading emails
- ☐ Writing emails

How often have you performed the following behaviors while driving in the last 30 days? on a scale of 0 to 4 (where 0 is never and 4 is always I drive)

	Never 0	1	2	3	4 Always I drive
13- Voice calling					
14- Reading texts					
15- Composing texts or texting					
16- Map browsing					
17- Social networking					
18- Reading emails					
19- Writing emails					

20- For what reason have you used your cell phone while driving in the last 30 days?

____

21- Have you ever had a phone-enhanced driving crash?”

- ☐ No
- ☐ Nearly
- ☐ Yes
