# Supplementary material for: “It’s okay because I’m just driving”: an exploration of self-reported mobile phone use among Mexican drivers
Source: PeerJ. 2024 Feb 23;12:e16899. doi: 10.7717/peerj.16899 (PMC10896083; doi:10.7717/peerj.16899)
Supplement: Supplemental Information 2 — The Spanish (i.e., originally applied) version of the root questionnaire used to conduct the research. [file peerj-12-16899-s002.pdf]

Appendix 1.
Questionnaire structure (Spanish version) (draft - researcher form)

Sección I.

1- ¿Puede decirme su ciudad de procedencia? ________________________

2- ¿Puede decirme su edad?________

3- Sexo:

- ☐ Hombre
- ☐ Mujer
- ☐ Otro
- ☐ Prefiero no decirlo

4- ¿Tiene licencia de conducción? (cuestión de filtro)

- ☐ Sí
- ☐ No

5- ¿Conduce al menos una vez a la semana? (cuestión de filtro)

- ☐ Sí
- ☐ No

Sección II.

6- ¿En qué grado considera que usar el teléfono móvil mientras conduce es arriesgado?, en una escala de 1 a 5 (donde 1 es en absoluto y 5 es de alto riesgo.

En absoluto 1	2	3	4	5 Alto riesgo

7- ¿Podría, por favor, indicar con qué frecuencia ha usado el teléfono móvil mientras conducía en los últimos 30 días?, en una escala de 0 a 4 (donde 4 es nunca y 4 es siempre que conduzco.

Never 0	1	2	3	4 Always I drive

¿Ha utilizado su teléfono móvil en las siguientes situaciones de tráfico en los últimos 30 días?

	Sí	No
8- Detenido en un semáforo		
9- Conduciendo con tráfico interminente		
10- Conduciendo a baja velocidad		
11- Conduciendo más rápido de 25 millas o 40 kilómetros por hora		

12- ¿Por qué razón/es ha usado el teléfono móvil mientras conducía en los últimos 30 días? (puede indicar más de una opción)

- ☐ Llamadas de voz
- ☐ Lectura de mensajes
- ☐ Redactar o enviar mensajes de texto
- ☐ Navegación por mapas
- ☐ Redes sociales
- ☐ Lectura de correos electrónicos
- ☐ Escribir correos electrónicos

¿Con qué frecuencia ha realizado los siguientes comportamientos mientras conducía en los últimos 30 días?, en una escala de 0 a 4 (donde 0 es nunca y 4 siempre que conduzco)

	Nunca 0	1	2	3	4 Siempre que conduzco
13- Llamadas de voz					
14- Lectura de mensajes					
15- Redactar o enviar mensajes de texto					
16- Navegación por mapas					
17- Redes sociales					
18- Lectura de correos electrónicos					
19- Escribir correos electrónicos					

20- ¿Por qué razón ha utilizado el teléfono móvil mientras conducía en los últimos 30 días?

____

21- ¿Ha tenido algún accidente al volante provocado por el teléfono?

- ☐ No
- ☐ Casi
- ☐ Sí
